# Laparoscopic versus open appendicectomy for complicated appendicitis: A prospective study

**DOI:** 10.4103/0971-9261.43803

**Published:** 2008

**Authors:** L. R. Padankatti, R. Kirthy Pramod, A. Gupta, P. Ramachandran

**Affiliations:** Department of Paediatric Surgery, Kanchi Kamakoti Childs Trust Hospital, 12-A, Nageshwara Road, Chennai, Tamilnadu, India

**Keywords:** Complicated appendicitis, laparoscopic appendicectomy, open appendicectomy

## Abstract

**Aims:**

The purpose of this study was to compare open versus laparoscopic appendicectomy (LA) in complicated appendicitis.

**Materials and Methods:**

We prospectively analyzed all children over a 2-year period who underwent appendicectomy in a single institution and found 30 cases of complicated appendicitis diagnosed on the table and confirmed by histopathology. These children were allocated randomly into Group 1 if they had LA and Group 2 if they had open appendicectomy (OA), solely based on surgeon assessment. The parameters assessed were duration of symptoms before surgery, use of postoperative parenteral analgesia, timing of initiation of feeds after surgery, postoperative complications and duration of postoperative stay.

**Results:**

Of the 30 patients with complicated appendicitis, 12 patients in Group 1 underwent LA and 18 patients in Group 2 underwent OA. The two groups were comparable for age and sex. Children in Group 1 were found to need less parenteral analgesia (two out of 12 versus six out of 18). Postoperatively, feeds were started earlier in Group 1 than in Group 2 (2.5 days versus 3.7 days). The average postoperative hospital stay was 5 days in Group 1 and 7.5 days in Group 2. There were only minor wound infections in Group1 as against two major complications in Group 2. There was no mortality in either group.

**Conclusions:**

In complicated appendicitis, laparoscopic approach carries definite advantages with less postoperative morbidity and hospital stay. It is a feasible and better alternative than the open approach in complicated appendicitis.

## INTRODUCTION

Acute appendicitis in children is the most common surgical emergency. Since the first laparoscopic surgery for appendicitis in 1983, it has been established as the gold standard surgery for simple appendicitis. Gangrenous/perforated appendix with or without periappendicular abscess, peritonitis and appendicular mass are accepted features of complicated appendicitis.[1] Over the recent years, the role of laparoscopic appendicectomy (LA) in complicated appendicitis has been gaining wider acceptance, but a few studies have reported increased postoperative morbidity with the same.[2] This study compares the open approach with the laparoscopic approach in complicated appendicitis, thereby bringing to light the feasibility of the laparoscopic approach as a viable alternative by identifying its advantages over the open procedure in complicated appendicitis.

## MATERIALS AND METHODS

The study is an ongoing single-institution randomized prospective analysis comparing open appendicectomy (OA) and LA for complicated appendicitis. Cases were allocated into open and laparoscopic groups based on surgeon preference. Cases were allocated to individual surgeons based on fixed admission days. The study began in May 2006 and data sheets were compiled on each patient. The following variables were included in the data sheet: age, sex, duration of symptoms before admission, clinical presentation, use of preoperative antibiotics and intravenous fluids, postoperative parenteral analgesia, time of establishment of oral feeds postoperatively, duration of postop stay and complications. For the purpose of this study, complicated appendicitis was defined as either a perforated or a gangrenous appendix with or without periappendicular pus, peritonitis or appendicular mass. The diagnosis of all patients was confirmed by histopathology postoperatively.

## RESULTS

Children were randomized into two groups. Group 1 consisted of all patients undergoing LA (n = 12). Group 2 consisted of all patients undergoing OA (n = 18). In Group1, nine children had perforation compared with 14 in Group 2. Also, in Group 1, three had mass compared with four in Group 2. The male to female ratio was 7:5 in Group 1 and 2:1 in Group 2. Average age in Group 1 was 7.5 years (range 2–10 years) and in Group 2 was 6.5 (range 4–14 years). All children in Group 1 were operated within 24 h of admission whereas three children in Group 2 received preoperative antibiotics and intravenous fluids for 2–3 days before surgery. All children in Group 1 underwent LA via three ports. The base of the appendix was secured with an endoloop and the peritoneal cavity was irrigated thoroughly. Two suction drains were placed in the pelvis and the paracolic gutter via port incisions. Standard OA was performed in all children in Group 2 through a muscle-splitting incision in the right iliac fossa. The base of the appendix was ligated and the stump was buried. The peritoneal cavity was irrigated thoroughly and the wound was closed. Peritoneal drainage was instituted with corrugated drain. Table 1 shows the significant comparison of variables in both the groups. Parenteral postop analgesia was required in two children in Group 1 and in six children in Group 2. Feeds were established in 2.5 days in Group 1 and 3.7 days in Group 2. The average duration of postop stay was 5.0 days in Group 1 and 7.5 days in Group 2. Two children in Group 1 had minor wound infection as against three children in Group 2. There was no major complication in Group 1 whereas one child in Group 2 had small bowel obstruction and fecal fistula needing relaparotomy, and one child had major wound dehiscence.

**Table 1:** Patient variables in the two groups

	Group 1 (LA)	Group 2 (OA)
Number (n)	12	18
With perforation	9	14
With mass	3	4
Male:female	7.5	2.1
Average age (years)	7.5	6.5
Operated within 24 h	12	15
Parenteral postop analgesia	2	6
Average starting of feeds	2.5	3.7
Average postop stay	5	7.5
Minor wound infection	1	3
Major complication	Nil	2

LA: Laparoscopic appendicectomy, OA: Open appendicectomy

## DISCUSSION

Laparoscopic surgeons usually recommend LA in patients with acute appendicitis, but controversy remains regarding the treatment of complicated appendicitis. Recent studies have suggested LA as a feasible alternative to OA in complicated appendicitis.[3] We have included patients with appendicular mass along with perforated appendices and appendicular abscesses as compared with previous studies where only perforations and abscesses have been compared.[4] This study is a prospective, randomized study that began 2 years ago. So far, 30 children have been enrolled with a diagnosis of complicated appendicitis. Randomization was based on the preference of the operating surgeons.

In LA, we prefer the 3-port technique. The use of three trocars during LA enables the removal of the appendix even during advanced stages of inflammation. The whole laparoscopic procedure is performed in situ without much mobilization of the caecum and distal part of the ileum to reduce the risk of tearing the inflamed wall of the caecum. Intestinal wall hematomas and postoperative bowel paralysis is less in comparison with the classic operation because of minimal bowel handling. Another advantage of the laparoscopic technique is better access to the appendix, especially if the appendix is found lying the pelvic cavity or in the subhepatic area.

In cases of periappendicular abscesses and peritonitis, laparoscopy enables more efficient pus evacuation and peritoneal cleaning. Laparoscopy also facilitates the dissection of caecum, small bowel and adherent omentum in cases of appendicular masses.

Children in Group 1 had less pain after surgery, more so in the early postoperative period (8–10 h), and reduced requirement for parenteral analgesia. Oral nonsteroidal antiinflamatory drugs (NSAIDs) were sufficient. This is in contradiction to previous studies, which recommended parenteral analgesia in all cases along with NSAIDs.[5].

Earlier initiation of feeds was possible in children with LA. The early return of bowel activity is probably attributable to lesser bowel handling and less elaborate adhesion release. An added factor could be lesser use of opioid analgesia, which prolongs postoperative ileus.

The duration of hospital stay was longer in patients with diffuse peritonitis and abscess formation than in patients with local peritonitis in both groups. This prolonged hospital stay was due to the need for intravenous antibiotics. Only minor wound infections were identified in patients with LA. In children who underwent standard appendicectomy, besides three cases of minor wound infections, there were two major complications. One was small bowel perforation with fecal fistula requiring relaparotomy and stay in hospital for almost 6 weeks. The other was a major wound dehiscence needing resuturing and stay in hospital for an extra week. There were no intraabdominal abscesses in either group, probably because all feculent and purulent material was removed and the abdominal cavity was rinsed with a large volume of saline.

Although previous studies on surgery for appendicular mass (open and laparoscopic) suggest that an interval appendicectomy be performed after a course of conservative treatment, we feel that with the laparoscopic approach it is possible to do primary appendicectomy because there is no morbidity with this procedure. In our series, both open and laparoscopic approaches were used to do primary appendicectomy in children with appendicular masses.

Although the number of patients enrolled in this study thus far is small, preliminary results show that our experience with LA for patients with complicated appendicitis has been encouraging. In conclusion, having found less morbidity with the laparoscopic approach than the open approach so far, we advocate the use of LA in children with complicated appendicitis.
